# Teachers’ perceptions of barriers and facilitators to peer play between children with autism spectrum disorder and typically developing peers in early childhood education: a research circle study in Austria

**DOI:** 10.1080/20473869.2023.2230410

**Published:** 2023-07-05

**Authors:** Johanna Linimayr, Line Lindahl-Jacobsen, Lisette Farias

**Affiliations:** 1 Institute of Occupational Therapy, Zurich University of Applied Sciences (ZHAW), Winterthur, Switzerland; 2 Department of Occupational Therapy, University College Absalon, Naestved, Denmark; 3 Division of Occupational Therapy, Department of Neurobiology, Care Sciences and Society (NVS), Karolinska Institutet, Stockholm, Sweden

**Keywords:** Peer play, autism spectrum disorder, teachers, mainstream classroom, research circle

## Abstract

Children with autism spectrum disorder (ASD) are increasingly being integrated into primary and secondary mainstream education. Yet, little is known about teachers’ challenges in supporting peer play for children with ASD in early childhood education. This study explores teachers’ perspectives on the barriers and facilitators to supporting peer play between children with ASD and their typically developing peers. Observations and research circle meetings were conducted with eight teachers from an urban area in Austria. Material from the meetings was analysed using qualitative content analysis and participants’ feedback. Findings illustrate how teachers perceive and manage multiple factors that influence peer play, including child-level factors (e.g. irritability to noise), peer and family factors (e.g. negative roles attributed to children with ASD), and institutional factors (e.g. large group sizes and lack of rooms without distractions). This study also highlights teachers’ ambivalence about safeguarding children’s participatory rights when encouraging children with ASD to engage in peer play when children with ASD need to disengage. This ambivalence is linked to the need for expanding the comfort zone of typically developing children by raising their awareness of diverse ways to interact and participate in play to support all children’s needs in inclusive education.

## Introduction

An increasing number of children with autism spectrum disorder (ASD) are accessing inclusive education (European Agency for Special Needs and Inclusive Education 2018, Lindsay *et al.* 2013) and spending most of their time with typically developing peers (O’Keeffe and McNally 2023). Although inclusive education aims to provide children with ASD opportunities to interact with typically developing peers, researchers highlight that mainstream education settings are struggling (Humphrey and Symes 2013, Lai *et al.* 2020; Lindsay *et al.* 2013). Little is known about teachers’ experiences working with children with ASD in mainstream education (Lindsay *et al.* 2013, Lindsay *et al.* 2014), and few studies have focused on teachers’ efforts supporting peer play in early inclusive education settings (Brodzeller *et al.* 2018, Zakai-Mashiach *et al.* 2021). This study addresses an important gap in the literature by exploring teachers’ experiences of barriers and facilitators to enabling peer play between children with ASD and their typically developing peers in early childhood education in Austria. This study is innovative due to the use of research circle methodology for data generation, providing a learning arena for participating teachers.

### Peer play

Play offers a naturalistic platform for supporting social communication and the inclusion of children with ASD in education settings (O’Keeffe and McNally 2023). Peer play experiences are seen as essential in children’s play development (Pyle and Alaca 2018) for their socialisation and participation (Wolfberg *et al.* 2015). For instance, peer play positively impacts children’s communication skills (Chapin *et al.* 2018), emotional regulation (Woodyatt and Rodger 2006), and social relationships and friendships (Wolfberg *et al.* 2015). Peer play has been described as another layer of play, hierarchically ranging in its symbolic complexity and its social dimensions (from solitary play to highly social role play) (Wolfberg 2019). However, play and peer play are being more critically discussed regarding their role in inclusive education (Pyle *et al.* 2017, Zosh *et al.* 2018). As such, play and peer play are considered part of a spectrum including equal facets of free play, guided play, games, and playful instructions (Zosh *et al.* 2018), which are relevant for children with ASD considering that they have different play styles and needs.

### Children with ASD

The play styles of children with ASD are often described as being qualitatively different from that of typically developing peers, showing less elaboration and more repetitive actions (Pinchover and Shulman 2016). Such difficulties often become more evident when children with ASD attend mainstream school due to increased social demands and expectations related to peer relations and interactions (Bauminger-Zviely 2014, Einfeld *et al.* 2018, O’Keeffe and McNally 2023). While play offers a naturalistic platform for supporting social communication and the inclusion of children with ASD in mainstream education settings (O’Keeffe and McNally 2023), their possibilities to participate in social activities with peers may be influenced not only by the heterogeneity of the child population but also by the amount of support and organized opportunities available at schools (Simpson *et al.* 2019; Taheri *et al.* 2016). This negatively influences their quality of life (Arias *et al.* 2018), communication (Chapin *et al.* 2018), and play development (Wolfberg *et al.* 2015). Research has shown that children with ASD are approximately 20 times more likely to be socially excluded (Humphrey 2008), indicating that involvement in peer play is concerningly lower than amongst their peers and that they may not always benefit from inclusive education settings (Simpson *et al.* 2018). Moreover, without appropriate support, the attempt to include children with ASD in mainstream education can result in further social isolation (Barber *et al.* 2016, Bass and Mulick 2007; Yang *et al.* 2003). Thus, children with ASD are in need of adult support to engage in and learn from play situations with peers (Simpson *et al.* 2018). To avoid social exclusion and the continuation of difficulties into adolescence, resulting in loneliness (Deckers *et al.* 2017) and bullying (Cappadocia *et al.* 2012, Humphrey and Hebron 2015), it is crucial to understand the challenges that educators may experience (Olsen *et al.* 2019).

### Teachers’ perspectives

Despite the benefits of inclusive education, more practice-based research with teachers is needed (Guldberg 2017, Parsons et al. 2018). Teachers have reported awareness of children with ASD’s behavioural difficulties and the use of strategies such as embracing children with ASD’s special interests and engaging in one-on-one time (Bolourian *et al.* 2022). Contrasting, they also feel unprepared to support children with ASD’s needs in mainstream education (Brodzeller *et al.* 2018, Lindsay *et al.* 2013, Olsen *et al.* 2019). Teachers are expected to meet the needs of all children, often with few or no guidelines or training to support children with ASD (Humphrey and Symes 2013, Lindsay *et al.* 2014, Olsen *et al.* 2019). Studies describing interventions and adaptations for children with ASD in inclusive education settings focus on meeting children’s learning needs and teaching social interaction skills by adapting the environment, materials, activity, and instructions (Brodzeller *et al.* 2018, Camargo *et al.* 2016), but few focus on supporting peer play, as different to peer training or the role of typical developing peers (Zakai-Mashiach et al. 2021). Moreover, research on teachers’ perspectives on working with children with ASD in mainstream classrooms is needed since many teachers lack training and have few or no guidelines on how to create an inclusive educational environment (Lindsay *et al.* 2013, Lindsay *et al.* 2014, Simpson *et al.* 2019). Yet, teachers are important and reliable sources with valuable experience to inform research about challenges in education settings (Chen *et al.* 2019, Potter 2015). Therefore, this study aimed to explore teachers’ perspectives on the barriers and facilitators to supporting peer play between children with ASD and their typically developing peers.

## Methods

Research suggests that teachers working with children with ASD in mainstream classrooms may not be equipped or have enough resources to employ inclusion strategies that respond to the needs of all children. To identify potential target areas for professional development and learn from each other’s experiences related to barriers and facilitators supporting peer play between children with ASD and their peers, the research circle methodology (Härnsten 1994) was chosen. This methodology benefits participants by expanding their knowledge through discussion, problem identification, and prioritization of relevant issues and possible strategies/solutions in collaboration with other participants. This collaboration and sharing of experiences can prompt new insights and opportunities for knowledge translation among the group. As such, this methodology reaches beyond sharing individual experiences by supporting participants’ development of competencies during the research process in which the object of study is the participants’ practice (Holmstrand *et al.* 2008, Åkerström and Brunnberg 2013, Östlund 2008, Wigg and Ehrlin 2018).

### Recruitment and participants

An information letter was distributed *via* email to six inclusive early education institutions and 14 local counselling service providers. Inclusion criteria were working in an inclusive early childhood education with at least one child between 3-6 years of age with an official diagnosis of ASD; having work experience for at least half a year; and showing a willingness to participate in all four research circle meetings. In total, 12 people responded, and four were excluded because they did not meet the inclusion criteria. The eight recruited participants were also asked to provide an information letter to the parents of children with ASD. Parents provided informed consent for their children to be observed in the educational environment. Teachers were not specifically trained to educate children with ASD and varied in experience and further education background (see Table 1: Participants’ Characteristics and Setting). Ethical approval was obtained from the ethics review board (blind information). The participants varied in age, education, years of experience, and professional role.

**Table 1.** Participants’ characteristics and setting.

Participants (P1-P8)	Age years	Education	Years of experience with children with ASD	Current role	Group size	Children’s status Regular: SEN (incl. ASD)	Adults in the group	Child with ASD Age Gender	
**P1**	33	early childhood education	<1	group leading teacher	13	11: 2 (2)	2 (1 teacher, 1 social pedagogue)	4 (twin 1)	girl
**P2**	24	social pedagogue	4	second teacher in the group	13	11: 2 (2)	2 (1 teacher, 1 social pedagogue)	4 (twin 2)	girl
**P3**	44	early childhood education, special education teacher, early interventionist	9	group leading teacher	15	11: 4 (1)	3 (1 teacher, 2 support staff)	5.5	boy
**P4**	52	early childhood education, after-school-care teacher	25	2nd teacher in the group	15	11: 4 (1)	2 (2 teachers)	5	girl
**P5**	43	early childhood education	4	group leading teacher	15	13: 2 (1)	3 (1 teacher, 2 support staff)	5	boy
**P6**	59	early childhood education	2	group leading teacher	15	12: 3 (3)	3 (1 teacher, 2 support staff)	4 4 4	boy boy girl
**P7**	54	early childhood education	25	group leading teacher	15	12: 3 (1)	3 (1 teacher, 2 support staff)	5.9	boy
**P8**	52	early childhood education	5	group leading teacher	14	11: 3 (1)	3 (1 teacher, 2 support staff)	4	girl

### The setting

In the Austrian education sector, teachers’ roles in early childhood education (ages 0-6) explicitly comprise enabling and actively guiding play and peer play activities to support children’s development (Hartel et al. 2019). The eight teachers involved in this study worked in seven different schools (two worked together). Their working conditions were comparable and typical for an inclusionary early childhood education setting in Austria. This means that inclusionary groups were located in the same building as several regular and/or special education groups, having their own main room and shared rooms with other groups (maximum group size 15 children). Each group had approximately four children with special education needs (SEN) status with two or three teachers per group composed of a leading teacher supported by assisting staff and/or another (special education) teacher. The time for more structured activities (e.g. circle time, learning activities) versus less structured activities (free play or centre time) varied in each group. The amount of time typically allocated for all children’s free play in this context varied between 2 and 5 h per day.

### Data collection

Data were collected in four research circle meetings where teachers discussed their experiences in depth. Each meeting lasted two hours, was held in German (the language of preference of the participants) and was facilitated by the first author and an assistant who were German-native speakers. The first author and assistant’s role was to lead every meeting by prompting questions and themes related to the study’s aim. The teachers met every two weeks with the first author and the assistant at the regional hospital. The structure of the meetings consisted of starting with open questions to prompt discussion among participants, then prioritising different aspects of their work, and reflecting upon related aspects in-between meetings. Participants were asked to bring photographs representing peer play barriers and facilitators to facilitate knowledge sharing. During research circle meetings, the first author visited participants’ workplaces for a one-hour observation. These observations were not part of the actual data collection process but were used to further discussions in the following meetings by sharing field notes. All meetings were audio-recorded and audiotaped for analysis.

### Data analysis

Data were analysed using Hsieh and Shannon’s (2005) recommendations for qualitative content analysis since it is useful for studies using research circle methodology (Åkerström and Brunnberg 2013) and descriptive research questions (Schreier 2020). After every research circle meeting, the audio-recorded discussions were transcribed verbatim and read multiple times by the first author to reach an in-depth understanding of the material. Data was then analysed using detailed line-by-line coding, and initial codes were labelled. Related codes were organised into preliminary categories that were presented to the participants at the beginning of every research circle meeting. These preliminary categories were used to build on previous discussions with participants’ feedback.

After the last research circle meeting, the first author continued analysing the preliminary categories that had been discussed with the participants, organising them into subcategories. This process entailed an iterative back-and-forth process between transcripts, the initial coding, preliminary categories, and participants’ feedback. Information highlighted by participants as related to barriers and facilitators of peer play was considered significant when defining each subcategory. Subcategories were then organised into preliminary categories, which were discussed among the authors and shared with participants *via* email. Participants’ feedback supported the definition of the final categories (see Table 2). A reflexive diary was used to keep notes and record decisions about why certain sentences were considered significant (Wilding and Galvin 2015). As a final step, quotes representing each final category were selected and translated from German to English (van Nes *et al.* 2010).

**Table 2.** Examples of initial codes, subcategories, categories, and quotes.

Examples of initial codes	Subcategories	Final categories	Examples of quotes
Joyful togetherness Linking-up Working on goals Attitudes towards participation Consistency Behaviours management	Teacher uses different strategies to facilitate peer play Teachers’ ambivalence about participation goals Teachers set small goals to see progression	Teachers’ involvement in overcoming peer play barriers	‘I think participation must be our primary goal. That’s what it’s all about in integration or inclusion or whatever you call it.’ (P3, RC-3)
Friendship and kindness Say please and thank you Articulate the other children’s feelings Negative roles	Practice kindness Role modelling of peers Verbalize feelings	Careful promotion of friendships and monitoring peer culture	‘The children experience him as a good play partner. He can be extremely kind. We practised it like this, we always say: ‘Dear …, please give me…’ And he always says please and thank you, and that pleases the children. (P7, RC-2)
Childcare ratio Distraction versus focus Group routines Team culture	Team culture and support Lack of understanding from other teachers Need to respond to all children’s needs	Lack of understanding from other teachers and management	‘Because it can be so exhausting and then you need someone who says: ‘Shall I continue? Do you want to take a break?’ Or You can go for a walk, I take over!’ (P4, RC-3)
Experiences from home Exposure to social experiences Refining collaboration	Parents rely on the so-called experts Parents roles Parents involvement and collaboration	Missing communication with parents to support peer play	‘Sometimes there is this thinking: You teachers, do! And the therapists, do! Maybe they do not feel competent enough or they just rely on the so-called experts.’ (P5, RC-4)

## Findings

Four main categories were identified. For traceability, quotes are marked with a participant number (e.g. P1 for Participant 1) and research circle number (e.g. RC-2 for the second research circle meeting).

### Teachers’ involvement in overcoming peer play barriers

Teachers described using various kinds of involvement to overcome peer play barriers; for example, proximal support by sitting beside the child with ASD, and providing physical or verbal prompting. They highlighted their role in building bridges between peers to overcome communication gaps, e.g. by supporting initiation or establishing turn-taking with verbal or physical prompting. Participant 1 described her ‘linking-up’ strategy:

When he looks angry: ‘Ooh! Look! How he is looking!’ It is about the small things, small behaviours, or changes in other children, that he cannot recognise because he is in his own world. Similarly, peers often do not understand either what is going on, and I verbalise it to them. (P1, RC-3)

By proactively using children’s interests, teachers try to increase children with ASD’s motivation to participate in peer play activities. They also adapt surroundings and group sequences to lower barriers to playful interactions (e.g. arranging small quiet play areas for two or three children or preparing selected playsets to make peer play more manageable). Some teachers set up specific peer play learning goals. To achieve these goals, teachers established daily routines, ritualised transitions (e.g. tidy-up song), provided highly structured adult-led play activities, and adjusted the amount and type of support to their progress. Yet, teachers expressed ambivalent feelings about why they should insist that children with ASD participate in peer play, especially when they were perceived as being easily stressed or showing uncomfortable, ambivalent, or indifferent feelings towards their peers. One teacher described this tension: ‘Why does he have to [participate]? Do we believe that he needs to be included? I question this.’ (P6, RC-3).

Teachers said they must constantly find stress-reducing strategies to prevent challenging behaviours and reduce peer play conflicts. For example, they use daily routines to meet children’s diverse needs by offering time for movement (e.g. jumping on a trampoline), calming activities (e.g. massage activities), or individual tools (e.g. waiting for a bag with favourite toys, picture cards to calm down). They also shared positive experiences with visual aids and regular group activities focusing on emotional regulation. Yet, teachers felt torn between attending to the needs of children with ASD and the rest of the group’s needs.

If something doesn’t work out as she wants it to, she starts crying. And it’s not only her crisis, but she also disturbs the play of the others, she interrupts it and stops it with her crying. So, this balance, that the others are OK in those situations is so difficult. (P4, RC-1)

### Careful promotion of friendships and monitoring of peer culture

The participants expressed that friendships between children with ASD and typically developing children were described as a trustworthy resource. Teachers highlighted peers’ roles as supporters. Some can become ‘very aware and knowledgeable about what [the child with ASD] needs’ *(P7, RC-2*) and, thus, can function as true facilitators during play. Positive peer culture and relationships were promoted by practising kindness among the children:

The children experience him as a good play partner. He can be extremely kind. We practised it like this, we always say: ‘Dear …, please give me…’ And he always says please and thank you, and that pleases the children. (P7, RC-2)

More mature peers were described as helpful for providing spontaneous guidance and support to children with ASD or by just being good communicators and players themselves. Yet, teachers explained that sometimes negative roles were attributed to children with ASD, making other peers reject, fear, or ignore them. To prevent conflicts and minimise mutual fears, teachers used strategies such as creating personal play space with a play carpet, reading a specific book about conflicts among peers, and the use of glove puppets in circle time to talk about feelings ‘because often [typically developing] peers do not understand either what is going on [with the child with ASD], so I verbalise it for them. (P1, RC-3). This verbalization support initiation and establishment of turn-taking. Sometimes, redirecting play into positive experiences was essential to minimise mutual fears:

He was just joyful, waving his arms. The others became afraid that he would disturb their building. So, I joined them and said, ‘Look, this tall building! You want to join in?’ And then we built together […] It is important to us that he sees his role expand and that he is not always the one who is crying, sleeping, or destroying things and running away. (P5, RC-2)

### Lack of understanding from other teachers and management

Teachers mentioned several contextual factors influencing peer play that were related to the institutional and the physical environment. Overall, big group sizes were perceived as overwhelming for the child with ASD, limiting their capacity to engage in peer play. Similarly, the lack of a physical and sensory room without distractions (e.g. big windows, noisy wide-open spaces) was perceived as problematic. Teachers needed to come up with solutions to make these spaces more appropriate for children with ASD by using diverse materials (sorted/labelled sets), sound-absorbing boxes to minimise noise, and arranging areas for movement, painting, or quiet reading:

The less stimulation, the better. Also, when the big room is overstocked, everyone is playing somewhere, and they get much more distracted. It is the opposite when you can offer spaces with low irritation. (P6, RC-4)

Some teachers described feeling a lack of institutional support, and understanding of their challenges from peers without children with ASD in their groups:

You need understanding from the other groups, from the management, from the whole team […] this develops over time, […] it is based on team development. Everybody needs to pull together. (P3, RC-4)

Teachers also highlighted the need for further education and constructive exchange with specialised external institutions.

### Missing communication with parents to support peer play

Though parents did not actively participate in peer play situations in early childhood education settings, teachers were convinced that they significantly influenced peer play. Some teachers described that ‘minimal contact with other children outside the early education setting’ *(P7, RC-1)* could have a negative impact on peer play performance in education settings, and therefore, more communication with parents was needed. Teachers highlighted parents’ roles as active promoters of their child’s development and as experts from whom they could learn more about the child’s preferences, interests, and strategies used at home.

I find it very effective when parents participate in the group, […] I think the child enormously benefits from this because you start aligning your handling […] parents are glad and appreciate this. […] And you build confidence and trust! (P4, RC-4)

Hence, missing communication with parents was sometimes perceived as frustrating, especially when teachers felt that they ‘do not pull together in the same direction, or when everybody reacts differently’ (P4, RC-1). To enhance the quality of family communications, teachers wished for more ‘time we do not have!’ (P8, RC-4). Though they value regular short parent conversations, they perceived having very little time for these activities in total (one hour/week for the whole group). If teachers anyhow take more time for this, they will need to ‘cut off’ (P1, RC-4) other responsibilities.

## Discussion

This study explored teachers’ perspectives on the barriers and facilitators to supporting peer play between children with ASD and their typically developing peers using a research circle methodology. Findings suggest that teachers perceive their role in peer play as fundamental. Previous research has shown that children with ASD require adult support to engage in peer play situations (Simpson *et al.* 2018). Teachers use several support strategies (e.g. situation-specific instructions, modelling, prompting) that have been identified in previous research as helping to support peer play (Brock *et al.* 2018, Chapin *et al.* 2018, Kuhaneck *et al.* 2020, Osborne *et al.* 2019). The teachers also showed their awareness of children with ASD’s emotional and behavioral difficulties, as sometimes children with ASD were easily stressed or showed uncomfortable, ambivalent, or indifferent feelings towards their peers. This awareness of core symptoms has been described as enabling teachers to embrace children with ASD’s needs through inclusion practices (Bolourian *et al.* 2022).

While more strength-based approaches have been shown to benefit children with ASD (Diener et al. 2015), it could be argued that perspectives focusing on individual resources and strengths might be less visible in this study. While teachers in this study highlighted the effects of positive peer culture, they seemed to approach typically developing peers more indirectly or on a group level rather than with specific individual goals to develop their skills as peer models, mediators, or peer buddies (Zakai-Mashiach *et al.* 2021). The role of peers must not be underestimated, especially in promoting participation in play activities (Anaby *et al.* 2013). Peers’ attitudes and knowledge influence the amount and quality of interactions (Bottema-Beutel *et al.* 2017, Petry 2018). Thus, expanding the comfort zone of typically developing children who tend to play with other typically developing peers can be an important strategy to support the quality of their peer interaction with children with ASD. Focusing on raising typically developing children’s awareness of diverse needs and ways of communicating or interacting would benefit not only children with ASD but the whole class group. Efforts invested in instructing and motivating peers to initiate social interaction seem to be more beneficial to foster social inclusion than working with a child with ASD on their difficulties (Bottema-Beutel *et al.* 2017, Kasari *et al.* 2016). Yet, most studies on peer play focus on either the child with ASD or their typically developing peers, while this study may indicate that integrating both approaches simultaneously would be relevant for practice. This may also include further investigating if and how typically developing peers’ benefit from playing with children with ASD.

Findings provide insights into teachers’ complex position, as they need to balance between fostering active participation and respecting individual preferences within play situations. Children with ASD may prefer spending time on their own, paying attention to specific interests or toys, while their typically developing peers prefer to play with each other (Bass and Mulick 2007). Teachers in this study described tensions when supporting a child with ASD that does not obviously show interest in peer play. It is possible that a child with ASD is happy being alone or needs to unwind from sensorial experiences and/or stress from social interactions (Olsen *et al.* 2019). In this case, some teachers argued that they failed the participation goal. Promoting participation while safeguarding children’s participatory rights seems to be a dilemma that should be carefully considered when developing inclusive early childhood education for children with ASD. Raising awareness of the difficulties that children with ASD may have, in both getting involved and expressing their right not to be included, is key (Biesta 2007).

Children with ASD may use alternative forms of non-social engagement to relax or unwind (Olsen *et al.* 2019), often perceived as withdrawal or inappropriate from a typical developmental view. This is problematic since the preferences and personal experiences of children with ASD should be considered when exploring peer play participation. Peer play perspectives of children with ASD and their typically developing peers are needed to expand teachers’ understanding of play and support their decision-making on when and how to support peer interaction (Calder *et al.* 2013). While it has been argued that play and learning constitute each other and shall not be understood as two different concepts (Pyle and Danniels, 2017), teachers may experience an ambiguity between the passive, non-directive enabling of free playtime and the active, directing, playful teaching (Pyle *et al.* 2017). Zosh *et al.* (2018) proposed to understand play as a spectrum incorporating facets of free play, guided play, games, and playful instructions. Rather than focusing on specific characteristics to distinguish free play from learning activities, it could be helpful to understand play and peer play along a continuum of child-led to teacher-led initiation and direction of activities, and the non-existence and existence of explicit learning goals. This understanding can support the self-determination of children with ASD and their choice about how, when, and for how long a child wants to participate in peer play.

Further, this study strengthens the assumption that children with ASD need individualised support to expand their possibilities within peer play situations. The importance of individualised support services for children with ASD has been reported in the literature (Harry *et al.* 2023, Thompson *et al.* 2019). Yet, these services are still insufficiently provided with appropriate learning and social environments (Lai *et al.* 2020). Missing communication with parents to support peer play was described as an important aspect of enabling individualised support. Teachers described parents as experts about their children with ASD’s preferences, interests, and strategies used at home. Therefore, coordination with parents about the direction in which support should be planned and delivered was deemed essential, together with institutional support for consultations with specialised professionals. German early childhood education teachers have reported the need for parent and case counselling support, more information about specific disability needs, and further education for the whole team (Wirts *et al.* 2018). To enhance individual support strategies for children with ASD in inclusive early childhood education settings, findings indicate that teamwork with parents, management, and specialist support is needed in mainstream environments.

## Implications for practice

Teachers experience themselves as fundamental facilitators but also perceive deficiency. Teachers require specialised support from management and other professionals to avoid frustration and expand their competencies. The amount of support children with ASD require to participate in peer play needs to be considered for developing inclusive early childhood education to meet children’s diverse needs (United Nations Educational, Scientific and Cultural Organization, 1994). More research is needed to find a balance between the children with ASD's need for and right to non-social activities, and efforts targeting to expand the child’s social comfort zone. Research exploring how typically developing children can expand their social comfort zone is also important, increasing children’s awareness of diverse needs and different ways in which children with ASD and other groups with learning difficulties communicate and engage in peer play.

## Limitations

The study was conducted with a small group of female teachers in an urban area in Austria. The experiences described by the participants only capture some experiences of inclusive education. Yet, this study provides valuable insights into the tensions that teachers face in mainstream education and can be relevant for developing more supportive environments and educational tools targeting peer play.

## Conclusion

Teachers use different strategies to overcome barriers and support children with ASD in social interactions, acknowledging the role of play in inclusive early education. Findings support the assumption that teachers have a fundamental role in supporting their peer play. Nevertheless, the findings also confirm that barriers to including children with ASD are still present in mainstream education. This study shows that institutional support is needed. Finally, a research circle methodology was shown to be useful in promoting knowledge-building, sharing, and learning from each other. It supported teachers’ involvement in the research process and may contribute to further implementation of research in practice.

## Data Availability

The data that support the findings of this study are available from the corresponding author, [LF], upon reasonable request.
